# Extragenital chlamydia infection among active-duty women in the United States Navy

**DOI:** 10.1186/s40779-019-0193-x

**Published:** 2019-01-29

**Authors:** Robert Deiss, Morgan Byrne, Sara M. Echols, Stephanie M. Cammarata, Lynda Potswald, Eduardo Gomez, Jennifer A. Curry, Eric Garges, Grace Macalino, Brian K. Agan, Mary F. Bavaro

**Affiliations:** 1 Infectious Disease Clinical Research Program, Department of Preventive Medicine and Biostatistics, Uniformed Services University of the Health Sciences, 11300 Rockville Pike, Rockville, MD 20850 USA; 2 The Henry M. Jackson Foundation for the Advancement of Military Medicine, Inc, 6720A Rockledge Drive, Bethesda, MD 20817 USA; 3 Division of Infectious Diseases, Naval Medical Center San Diego, 34800 Bob Wilson Drive, Ste 201, San Diego, CA 92134 USA; 4 32nd Street Branch Medical Clinic, Naval Medical Center San Diego, 2450 Craven St, San Diego, CA 92104 USA; 5 Division of Tropical Medical Health, Department of Preventive Medicine and Biostatistics, Uniformed Services University of the Health Sciences, 4301 Jones Bridge Road, Bethesda, MD 20814-4799 USA; 6 Marimac Insight, LLC, Simpsonville, MD USA

**Keywords:** Chlamydia, Military, Risk behavior, Sexually transmitted infection

## Abstract

**Background:**

Pharyngeal and anorectal reservoirs of gonorrhea (GC) and chlamydia (CT) are increasingly recognized among heterosexual women. While a number of studies performed at sexually transmitted disease (STD) clinics have found a high prevalence of extragenital GC/CT infection, such screening is typically not offered during routine primary care visits for women. We sought to define the prevalence of and factors associated with extragenital GC/CT among women in the U.S. Navy.

**Methods:**

We recruited servicewomen stationed in San Diego, California, between the ages of 18 and 25 who presented for an annual physical exam between January and September, 2017. Nucleic acid amplification testing was performed on swabs collected at endocervical, oropharyngeal and anorectal sites to assess the presence of GC/CT. An anonymous behavioral questionnaire was also administered to characterize sexual risk. Descriptive statistics were used to compare women with and without a prior history of any sexually transmitted infection (STI) (self-report) along with a current GC/CT diagnosis. This study was approved by the Institutional Review Board of the Uniformed Services University of Health Sciences.

**Results:**

Of the 75 patients who were approached, 60 subjects were enrolled in the study, including white 20 (33.3%), black/African American 18 (31.0%), Hispanic/Latina 13 (21.7%) and Asian/Pacific Islander 9 (15.5%) women. Among all the women, six (10.0%) were diagnosed with CT infection, all via endocervical exam. Of these, five (8.3%) had concurrent anorectal infection, including two cases (3.3%) accompanied by pharyngeal infection. Of the subjects, 15 (25.0%) reported anal intercourse in their most recent sexual encounter, most of which was condomless (13/15, 86.7%). A high number of women who reported sex with a casual male partner (19/45, 42.2%) reported rarely or never using condoms; last, 41.7% consuming at least 3 drinks on a typical drinking day, and one-third of the reported drinking more than once per week.

**Conclusions:**

We found a high prevalence of anorectal CT infection, although no infections were detected without concurrent endocervical involvement. Nonetheless, the high prevalence of condomless anal intercourse reported by participants argues for further study and ongoing consideration of extragenital screening among high-risk patients. Behavioral interventions are also warranted given the high prevalence of sexual and related risk factors.

## Background

Women serving in the United States (U.S.) military are often considered a high-risk population for the acquisition of sexually transmitted infections (STIs). Military demographics contribute to this higher incidence, as younger age, education at the high school (as opposed to college) level, residence in high-prevalence areas and African American race are commonly described as risk factors for STIs, and several of these demographics are overrepresented in the military [1–3]. In addition, a number of sexual risk behaviors, particularly inconsistent condom use or having multiple sexual partners, are highly prevalent among military servicewomen [4–6]. The end result is that STI rates in the military commonly exceed age-matched rates in the general population [4, 7–10].

Increased attention has been given to extragenital reservoirs of gonorrhea (GC) and chlamydia (CT) among heterosexual women. In two U.S. studies of women tested at urban sexually transmitted disease (STD) clinics, the prevalence of anorectal GC/CT ranged between 3.0 and 6.0% and 8.7 and 13.0%, respectively [11, 12]. Pharyngeal and anorectal GC/CT infections are typically asymptomatic; therefore, exclusive reliance on urogenital testing in high-risk populations has been shown to miss a number of extragenital GC/CT infections [11–14]. Some of these infections may later become symptomatic or facilitate transmission to other individuals who develop more serious infections. Extragenital screening can also promote counseling and awareness that STI transmission is still possible through sexual practices where condoms are less commonly used. Treatment for asymptomatic infections may also differ based on the pathogen and location. For these reasons, extragenital screening of heterosexual women is increasingly advocated, particularly as infections are often detected even without self-reported oral/anal intercourse [15, 16].

In this pilot study, we sought to examine the prevalence of asymptomatic extragenital GC/CT infection and associated patterns of sexual behavior among young women in the U.S. Navy who are not otherwise identified as at high risk for STIs. A second exploratory objective was to assess the feasibility and acceptance of routine extragenital testing in a primary care setting.

## Methods

Between January and September, 2017, we recruited women between the ages of 18 and 25 who presented to their primary care clinic for an annual exam, as is required of women in the military. We excluded women who were experiencing any symptoms related to GC/CT infection, including urethritis, dyspareunia, pharyngitis and pelvic, abdominal or rectal pain. Prior to clinical examination, the consenting subjects underwent provider-administered endocervical, pharyngeal and rectal swab testing, and the samples were analyzed via nucleic acid amplification testing (Hologic; San Diego, CA). In addition, the women completed an anonymous paper self-administered questionnaire to describe drug/alcohol use and sexual behavior; these surveys were linked to clinical data through use of a single participant identifying number (PIN) without personal identifying information. Self-reported race and ethnicity were also elicited, given the association between STI prevalence and membership of certain demographic groups. All study activities took place at an on-base primary health care clinic in San Diego, California. All patients provided informed consent for the study. The study was approved by the Institutional Review Board of the Uniformed Services University of Health Sciences.

Descriptive statistics were used to compare women with and without a prior history of any STI (self-report) and current GC/CT diagnosis. Nonparametric tests were used to compare medians; Chi-square and Fisher-exact tests were used to compare proportions between groups. Analyses were conducted using SAS software (version 9.3; Cary, NC).

## Results

A total of 75 patients were approached for study participation. Of these, 10 patients were excluded for reporting symptoms consistent with STIs, and 5 opted not to participate, yielding a final sample of 60 women. Demographic characteristics are presented in Table 1. The median age of all participants was 23 years old (interquartile range [IQR 21, 24]) and included 20 (33.3%) white, 18 (31.0%) black/African American, 13 (21.7%) Hispanic/Latina and 9 (15.5%) Asian/Pacific Islander women. One-third of the women (*n* = 20) reported a lifetime history of sexual assault.

**Table 1:** Demographic characteristics by diagnosis of gonorrhea (GC) or chlamydia (CT), or presence of any prior sexually transmitted infection (STI) by self-report

Item	Overall (*n* = 60)	Current GC/CT^a^			Prior STI		
		No (*n* = 54)	Yes (*n* = 6)	*P*-value	No (*n* = 41)	Yes (*n* = 19)	*P*-value
Age (Median, interquartile range)	23 (21,24)	23 (21,24)	20.5 (19,23)	0.022	22 (21,24)	23 (22,24)	0.063
Median years of military service (interquartile range)	2.7 (1.7,3.9)	2.8 (1.8,3.9)	1.6 (1.3,2.7)	0.171	2.6 (1.7,3.9)	2.82 (1.6,3.9)	0.799
Education (*n* (%))				0.738			0.901
High school diploma or GED	26 (43.4)	22 (40.7)	4 (66.7)		18 (43.9)	7 (36.8)	
Some college or Associate degree	23 (38.3)	21 (38.9)	2 (33.3)		14 (34.2)	9 (47.4)	
Currently enrolled in college	3 (5.0)	3 (5.6)	0 (0)		2 (4.9)	1 (5.3)	
4-year college degree	8 (13.3)	8 (14.8)	0 (0)		6 (14.6)	2 (10.5)	
Race/Ethnicity (*n* (%))				0.840			0.875
White	20 (33.3)	17 (31.5)	3 (50.0)		15 (36.6)	5 (26.3)	
Black/African American	18 (31.0)	16 (29.6)	2 (33.3)		12 (29.3)	6 (31.6)	
Hispanic/Latino	13 (21.7)	12 (22.2)	1 (16.7)		8 (19.5)	5 (26.3)	
Asian/Pacific islander	9 (15.5)	9 (16.7)	0 (0)		6 (14.6)	3 (15.8)	
Rank (*n* (%))				0.436			0.648
Enlisted	55 (91.7)	49 (90.7)	6 (100)		38 (92.7)	17 (89.5)	
Officer	5 (8.3)	5 (9.3)	0 (0)		3 (7.3)	2 (10.5)	
Current marital status (*n* (%))				0.825			0.182
Never married	37 (61.7)	32 (59.3)	5 (83.3)		26 (63.5)	11 (57.9)	
Married/Life partner	17 (28.3)	16 (29.6)	1 (16.7)		13 (31.7)	4 (21.0)	
Married/Life partner (separated)	4 (6.7)	4 (7.4)	0 (0)		1 (2.4)	3 (15.8)	
Divorced	2 (3.3)	2 (3.7)	0 (0)		1 (2.4)	1 (5.3)	
Victim of sexual assault, ever (*n* (%))				0.325			0.466
No	35 (58.3)	31 (59.6)	4 (66.7)		22 (53.7)	13 (68.4)	
Yes	20 (33.3)	19 (36.5)	1 (16.7)		15 (36.6)	5 (26.3)	
Prefer not to answer	3 (5.0)	2 (3.9)	1 (16.7)		3 (7.3)	0 (0)	
Time deployed (*n* (%))				0.663			0.400
None	35 (58.3)	31 (57.4)	4 (66.7)		22 (53.7)	13 (68.4)	
1–2 times	25 (41.7)	23 (42.6)	2 (33.3)		19 (46.4)	6 (31.6)	
Last deployed (*n* (%))				0.608			0.5812
Within the past 12 months	6 (20.7)	6 (23.1)	0 (0)		5 (23.8)	1 (12.5)	
12 and 24 months ago	14 (48.3)	12 (46.1)	2 (66.7)		11 (42.4)	3 (37.5)	
24^+^ months ago	5 (17.2)	5 (19.2)	0 (0)		3 (14.3)	2 (25.0)	
Prefer not to answer	4 (13.8)	3 (11.5)	1 (33.3)		2 (9.5)	2 (25.0)	

Missing values are not included in the table. ^a^All infections were CT. *GED* General educational development. *P*-values derived from the Kruskal-Wallis test for continuous variables and the chi-squared or Fisher exact test with Monte Carlo estimation for categorical variables

With respect to sexual behavior (Table 2), 58 (96.7%) reported being sexually active over the prior year and had a regular sexual partner, and 30 (50.0%) reported two or more partners in the last 6 months. In addition, 23 (38.3%) of the women reported having had a casual sexual partner over the last 6 months; of the women who reported sex with a casual partner at any point in time, 19 of 45 (42.2%) reported that they rarely or never use condoms. With respect to alcohol use, 25 (41.7%) of all the women reported consuming at least 3 drinks on a typical drinking day, and 20 (33.3%) reported drinking more than once per week. When asked about their most recent sexual encounter, 56 (93.3%) of the women reported vaginal sex with a male partner while 57 (95.0%) and 15 (25.0%) of women reported oral and anal sex, respectively. Most anal intercourse 86.7% (13/15) was condomless in these encounters.

**Table 2:** General sexual and alcohol-related risk behaviors by prior sexually transmitted infection (STI) (n (%))

Item	Overall (*n* = 60)	Current or prior STI		
		No (*n* = 37)	Yes (*n* = 23)	*P*-value^a^
Vaginal sex with male partner				0.978
Never	1 (1.7)	1 (2.7)	0 (0)	
One time or a few times a year	9 (15.0)	5 (13.5)	4 (17.4)	
Monthly	6 (10.0)	3 (8.1)	3 (13.0)	
Once a week	6 (10.0)	4 (10.8)	2 (8.7)	
A few times a week	35 (58.3)	22 (59.5)	13 (56.5)	
Daily	2 (3.3)	1 (2.7)	1 (4.4)	
Prefer not to answer	1 (1.7)	1 (2.7)	0 (0)	
Condom use during vaginal sex				0.195
Never	30 (50.0)	15 (40.6)	15 (65.2)	
Rarely	8 (13.3)	6 (16.2)	2 (8.8)	
Sometimes	8 (13.3)	5 (13.5)	3 (13.0)	
Usually	8 (13.3)	5 (13.5)	3 (13.0)	
Always	6 (10.0)	6 (16.2)	0 (0)	
Anal sex				0.284
Never	41 (68.3)	25 (67.6)	16 (69.6)	
One time or a few times a year	17 (28.3)	12 (32.4)	5 (21.7)	
Monthly	1 (1.7)	0 (0)	1 (4.4)	
A few times a week	1 (1.7)	0 (0)	1 (4.4)	
Condom anal sex				0.931
Never	19 (63.3)	12 (57.1)	7 (77.8)	
Rarely	3 (10.0)	2 (9.5)	1 (1.1)	
Sometimes	2 (6.7)	2 (9.5)	0 (0)	
Usually	1 (3.3)	1 (4.8)	0 (0)	
Always	3 (10.0)	2 (9.5)	1 (1.1)	
Prefer not to answer	2 (6.7)	2 (9.5)	0 (0)	
Contraception use				0.571
No	18 (30.0)	10 (27.0)	8 (34.8)	
Yes	42 (70.0)	27 (73.0)	15 (65.2)	
Have main sexual partner				0.730
No	2 (3.3)	1 (2.7)	1 (4.3)	
Yes	58 (96.7)	36 (97.3)	22 (95.7)	
Gender of main partner				0.581
Male	54 (90.0)	33 (89.2)	21 (91.3)	
Female	4 (6.7)	3 (8.1)	1 (4.3)	
Main partner, active duty military				0.243
No	16 (26.7)	12 (32.4)	4 (17.4)	
Yes	42 (70.0)	24 (64.9)	18 (78.3)	
Sexual partners last 6 months				0.471
None	9 (15.0)	5 (13.5)	4 (17.4)	
One	12 (20.0)	8 (21.6)	4 (17.4)	
2–4	27 (45.0)	15 (40.5)	12 (52.2)	
5–8	3 (5.0)	3 (8.1)	0 (0)	
Prefer not to answer	1 (1.7)	1 (2.7)	0 (0)	
Casual number of sexual partners				0.791
None	28 (53.9)	18 (48.7)	10 (43.5)	
Some	5 (9.6)	3 (8.1)	2 (8.7)	
Half	5 (9.6)	2 (5.4)	3 (13.0)	
Most	5 (9.6)	4 (10.8)	1 (4.3)	
All	8 (15.4)	6 (16.2)	2 (8.7)	
Prefer not to answer	1 (1.9)	1 (2.7)	0 (0)	
Frequency of condom use with casual male partner				0.699
Never	16 (32.0)	9 (24.3)	7 (30.4)	
Rarely	3 (6.0)	2 (5.4)	1 (4.3)	
Sometimes	5 (10.0)	2 (5.4)	3 (13.0)	
Usually	5 (10.0)	4 (10.8)	1 (4.3)	
Always	16 (32.0)	12 (32.4)	4 (17.4)	
Prefer not to answer	5 (10.0)	3 (8.1)	2 (8.7)	
Frequency of alcohol consumption, last 6 months				0.157
4 or more times a week	2 (3.4)	0 (0)	2 (8.7)	
2–3 times a week	18 (30.5)	11 (29.7)	7 (30.4)	
2–4 times a month	18 (30.5)	12 (32.4)	6 (26.1)	
Once a month or less	15 (25.4)	9 (24.3)	6 (26.1)	
I did not drink alcohol in the past year	4 (6.8)	4 (10.8)	0 (0)	
I have never drunk any alcohol in my life	1 (1.7)	0 (0)	1 (4.4)	
Prefer not to answer	1 (1.7)	0 (0)	1 (4.4)	
Alcohol drinks typical day, last 6 months				0.929
10 or more drinks	3 (5.2)	2 (5.7)	1 (4.4)	
7–9 drinks	1 (1.72)	0 (0)	1 (4.4)	
5–6 drinks	5 (8.62)	3 (8.6)	2 (8.7)	
3–4 drinks	16 (27.59)	9 (25.7)	7 (30.4)	
1–2 drinks	27 (46.55)	17 (48.6)	10 (43.5)	
Prefer not to answer	6 (10.34)	4 (11.4)	2 (8.7)	
Sex after alcohol				0.602
Never	13 (21.7)	9 (24.3)	4 (17.4)	
Rarely	19 (31.7)	11 (29.7)	8 (34.8)	
Sometimes	18 (30.0)	12 (32.4)	6 (26.1)	
Often	6 (10.0)	4 (10.8)	2 (8.7)	
Every time	2 (3.3)	0 (0)	2 (8.7)	
Prefer not to answer	2 (3.3)	1 (2.7)	1 (4.4)	
Last sexual encounter				
Engaged in vaginal sex				–
No	4 (6.7)	3 (8.1)	0 (0)	
Yes	56 (93.3)	34 (91.9)	23 (100)	
Performed oral sex on partner				–
No	1 (1.7)	1 (2.7)	0 (0)	
Yes	57 (95.0)	34 (91.9)	23 (100)	
Engaged in anal sex				0.873
No	45 (75.0)	31 (75.6)	14 (73.7)	
Yes	15 (25.0)	10 (24.4)	5 (26.3)	
Used condoms^b^				–
No	13 (86.7)	7 (77.8)	6 (100)	
Yes	2 (13.3)	2 (22.2)	0 (0.0)	

Missing values are not included in the table. ^a^*P*-values derived from Kruskal-Wallis test for continuous variables, and chi-squared or Fisher exact test with Monte Carlo estimation for categorical variables; ^b^*n* = 15 overall, *n* = 9 for women with no history of STI, *n* = 6 for women with current/prior STI

Six women (10.0%) were diagnosed with current chlamydia infection, all via endocervical exam, including one case without extragenital infection. Concurrent anorectal infection was noted among five women (8.3%), including two cases accompanied by pharyngeal infection (3.3%). Pharyngeal infection without genital infection was not observed.

## Discussion

We found a high prevalence of chlamydia among military servicewomen, particularly among those with a prior history of an STI, consistent with the findings of larger studies [2, 3]. Neither pharyngeal nor rectal GC/CT were observed without endocervical infection, although five of six infections involved extragenital sites. The common practices of oral and anal intercourse, as reported by study participants, demonstrate a clear, ongoing risk for extragenital GC/CT infection.

Our lack of additional case-finding via extragenital screening differs from several large studies. A review of 4402 women reporting extragenital exposures at two Baltimore STD clinics found that urogenital-only testing would have missed 30.3% of GC and 13.8% of CT infections [12]. In the United Kingdom, an audit of GC/CT screening at an STD clinic found that 11% of pharyngeal CT cases (7/62) and 29% (2/7) would have otherwise been undiagnosed without extragenital testing [13]. It is likely that our small sample size contributed to this difference, along with the inherent differences between individuals seeking care at a primary care versus STD clinics.

Given the high concordance of anorectal and urogenital CT infection, it seems likely that a larger sample may have uncovered cases of exclusive anorectal infection, even as we did find a high proportion of concurrent infection. Likewise, in their retrospective review of STD clinic visits in Columbus, Ohio, Bazan et al [11] found that rates of anorectal and urogenital GC/CT were similar, and 19% of the women had anorectal infection alone. Findings such as these have directed many programs toward broader screening. For instance, a review of STI clinic records in Amsterdam found no significant difference in the prevalence of anorectal CT among 192 women (10.4%) who underwent universal screening compared with 4405 women who underwent selective testing (9.5%). As a result, the authors argue that universal screening should be favored in light of the notion that many women may not automatically identify as being at-risk for extragenital GC/CT infection [14]. At non-STI clinics such as ours, a patient-directed approach may nonetheless be reasonable.

To our knowledge, this is the first description of patterns of anal intercourse among military women. Nearly one-third of the women reported anal intercourse within the past year, and most of these encounters were condomless. Significant overlap was also observed in the type of sex reported at the last sexual encounter, as has been observed in other studies [17]. Of course, subjects may have opted for extragenital testing based on their perceived risk, and the true extent of this sexual practice may be lower; nevertheless, considerable risk is already evident from these data alone. Thus, education and interventions to decrease the incidence of STIs should also emphasize the potential for anorectal STIs, along with the increased risk for human immunodeficiency virus (HIV) acquisition from localized tissue trauma during condomless anal intercourse.

Our behavioral questionnaire also elucidated a high volume and frequency of alcohol consumption, with 41.7% of the women consuming three or more drinks on a typical drinking day. These findings are consistent with other reports of alcohol-related risk among military women. In a study of 3083 active-duty personnel, 38% reported binge drinking (> 4 drinks) over the last 30 days [18]. O’Rourke et al [5] found a similar prevalence of binge drinking (33%) and found that it was associated with a decreased likelihood of contraception use, including condoms. We did not obtain detailed histories on situational alcohol use, but prior research among Navy servicewomen has found that they may use binge drinking as a way to fit in with male counterparts, which can leave them vulnerable to unwanted sexual encounters [19].

Although not associated with GC/CT infection, the high prevalence of lifetime sexual assault in our study (33.3%) is noteworthy. The most recent Department of Defense annual report on sexual assault found a 10% increase in the number of reported assaults between 2016 and 2017, though related surveys have found an actual decrease between 2012 and 2016 of 43% [20]. We did not ask whether the assault occurred in or outside the military; nevertheless, these data have implications for the physical health, psychological well-being, and STI exposure of women. For instance, one study found an association between sexual assault and cytologic abnormalities among 999 female veterans, 62% of whom reported a lifetime history of sexual assault [21]. Screening for sexual assault in clinical care encounters and research studies of women’s health is therefore warranted.

This study had several limitations. As a cross-sectional study, causal inferences cannot be inferred. All questionnaire-related data were collected by self-report and may have been subject to social desirability bias. The survey, however, was anonymous and self-administered, and thus these effects were likely minimal. In any event, we report a high prevalence of socially undesirable behaviors (e.g., condomless anal intercourse), which should have biased data in the opposite direction. Finally, as noted above, our small sample size permitted a more robust comparison of trends associated with extragenital GC/CT infections.

When part of routine care, we found that extragenital STI testing was widely accepted by women, as evidenced by the rate of recruitment into our study. Providers in our study required only minimal training for the performance of pharyngeal and rectal swabs, which themselves are increasingly self-performed [22]. While recommendations for STI screening in routine primary care are designed for use in the general population, our findings suggest that military women may have increased risk for STIs, and screening criteria may need to be more inclusive. The widespread exposures and significant extent of anorectal infection may provide justification for wider adoption and study of extragenital GC/CT testing among women in the U.S. military.

## Conclusions

We found a high prevalence of chlamydia in our sample of active-duty women in the U.S. Navy, and while neither pharyngeal nor rectal infections were observed without endocervical infection, five of the six diagnosed cases involved extragenital sites. The high prevalence of condomless anal intercourse reported by participants indicates a need for further study and ongoing consideration of extragenital GC/CT screening among high-risk patients. Behavioral interventions are also warranted given the high prevalence of sexual and related risk factors.

## Abbreviations

CT: Chlamydia
GC: Gonorrhea
IQR: Interquartile range
STD: Sexually transmitted disease
STI: Sexually transmitted infection
U.S.: United States
